# Coherent modulation of chiral nonlinear optics with crystal symmetry

**DOI:** 10.1038/s41377-022-00915-4

**Published:** 2022-07-08

**Authors:** Yi Zhang, Xueyin Bai, Juan Arias Muñoz, Yunyun Dai, Susobhan Das, Yadong Wang, Zhipei Sun

**Affiliations:** 1 Department of Electronics and Nanoengineering, Aalto University, 02150 Espoo, Finland; 2 QTF Centre of Excellence, Department of Applied Physics, Aalto University, 02150 Espoo, Finland; 3 Advanced Research Institute of Multidisciplinary Sciences, Beijing Institute of Technology, 100081 Beijing, China

**Keywords:** Nonlinear optics, Photonic devices

## Abstract

Light modulation is of paramount importance for photonics and optoelectronics. Here we report all-optical coherent modulation of third-harmonic generation (THG) with chiral light via the symmetry enabled polarization selectivity. The concept is experimentally validated in monolayer materials (MoS_2_) with modulation depth approaching ~100%, ultra-fast modulation speed (<~130 fs), and wavelength-independence features. Moreover, the power and polarization of the incident optical beams can be used to tune the output chirality and modulation performance. Major performance of our demonstration reaches the fundamental limits of optical modulation: near-unity modulation depth, instantaneous speed (ultra-fast coherent interaction), compact footprint (atomic thickness), and unlimited operation bandwidth, which hold an ideal optical modulation solution for emerging and future nonlinear optical applications (e.g., interconnection, imaging, computing, and quantum technologies).

## Abstract (web summary)

Coherent all-optical modulation of nonlinear optics with chiral light in symmetrical crystals is achieved with ultra-fast speed (~ fs), unlimited bandwidth, perfect modulation depth (~100%), and compact footprint (atomic thickness).

## Graphical abstract

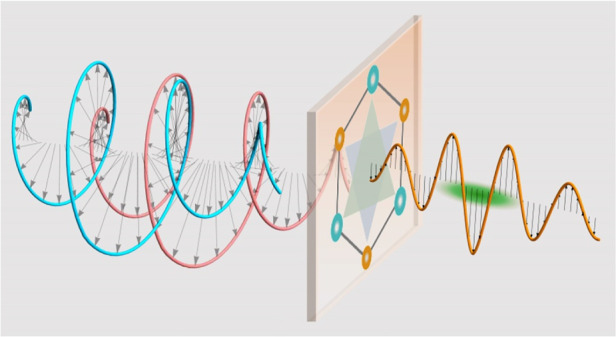

## Introduction

Optical modulation plays an important role in modern photonics and optoelectronics, such as optical interconnection, imaging, computing, and quantum technologies^1–3^. To meet the increasing demand for high-performance optical signal processing in the information era, optical modulators with compact footprint, ultra-fast speed, high efficiency, and broadband operations are highly desired^1^. Among them, nonlinear optics based optical modulation attracts much attention, due to the performance advantages of the coherent light-matter interaction^4,5^. For example, all-optical modulations utilizing third-order nonlinear optics (e.g., saturable absorption^6^ and optical Kerr effect^7^) have been demonstrated for various applications, such as ultra-fast lasers^6^ and phase shifters^7^. As a common and widely used third-order nonlinear optical process, third-harmonic generation (THG) is always present in a large range of materials (such as silica^8^, metamaterials^9,10^, and 2D materials^11–16^), in contrast to the second-harmonic generation (SHG) that only exists in non-centrosymmetric materials^5^. Recently, all-optical modulations of THG in hybrid micro-systems^17,18^, gold metasurfaces^19^, and 2D layered materials^20,21^ have been explored. However, these all-optical modulation methods typically involve carrier relaxation, and thus are naturally incoherent with a relatively slow modulation speed (>~ps), a typical modulation depth of ~90%^20,21^, and polarization non-selectivity. Therefore, novel all-optical modulation strategies with high performance in speed and modulation depth, and polarization selection capability are thus of great importance.

As a fundamental property of the electromagnetic field, optical chirality, embodied by left-handed (*σ*^−^) and right-handed (*σ*^+^) circular polarization, has been long employed to explore chiral light-matter interaction both in linear optics (e.g., circular dichroism^22^) and nonlinear optics (e.g., chiral harmonic generations^23,24^). Chiral nonlinear optics is becoming increasingly important in the rapidly growing fields of optics and optoelectronics^25,26^. Accordingly, the efficient control of chiral nonlinear optical responses is desirable for practical applications, but still remains elusive. Here we report all-optical coherent modulation of THG with chiral light via the polarization selectivity induced by *D*_3*h*_ symmetry of monolayer MoS_2_. All-optical modulation with performance reaching fundamental limits (a modulation depth of ~100%, ultra-fast speed of <~130 fs, and unrestrained operation bandwidth) is demonstrated with a minimum pulse energy of ~50 pJ. Moreover, the modulation performance can be highly tuned by the incident light power and polarization. Our approach depends only on the symmetry of the crystal structure and thus can be applied to other materials and structures with similar symmetries. This demonstration paves the way toward high-performance all-optical modulation, and also offers a new alternative for ultra-short pulse characterization different from current methods based on non-centrosymmetric materials.

## Results

### Modulation concept of chiral nonlinear optics

**Fig. 1:**
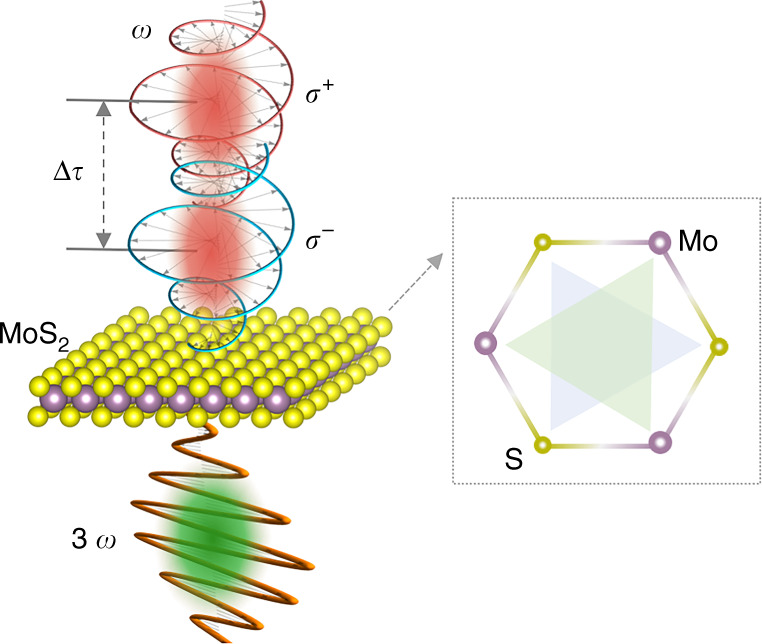
Schematic illustration of coherent modulation of THG with chiral light. The spatially overlapped two incident beams with opposite circular polarization states (*σ*^−^ and *σ*^+^) at the same frequency of *ω* lead to modulated THG at a frequency of 3*ω* as a function of the time delay (Δ*τ*) in monolayer MoS_2_ (acting as modulator). Inset is the top view of the crystal structure of monolayer MoS_2_, and the triangles inside imply the *D*_3*h*_ symmetry.

We present the operation principle of modulating THG in monolayer MoS_2_ by incorporating two input pump beams with identical frequencies. A typical case of the two input beams with opposite chirality is schematically illustrated in Fig. 1. Different input polarization states (e.g., a linearly- and a circularly-polarized incident beam) are also studied, as discussed later. The two spatially overlapped input beams with a time delay Δ*τ* are incident onto the operating material (MoS_2_). The THG in the material is thus modulated by the time delay (Δ*τ*) and reaches the maximum at Δ*τ* = 0 (i.e., the simultaneous presence of two incident beams) due to the coherent modulation process. Our modulation mechanism is based on the material symmetry and polarization selectivity of the third-order susceptibility. Taking monolayer MoS_2_ (belonging to the *D*_3*h*_ symmetry group^27,28^ as shown in the inset of Fig. 1) as an example, we analyze the underlying physics by deriving the third-order nonlinear process of THG. The third-order nonlinear polarization **P**^(3*ω*)^ can be obtained via $P_i^{\left( {3\omega } \right)} = {\it{\epsilon }}_0\chi _{ijkl}^{(3)}E_jE_kE_l$, where ${\it{\epsilon }}_0$ is the permittivity of free space, $\chi _{ijkl}^{(3)}$ is the third-order susceptibility (detailed form given in Section 1 of Supplementary Information (SI)), and subscripts *i(j)*, *k*, and *l* run over *x*, *y*, and *z* coordinates, *E*_*j*(*k,l*)_ denotes the electric component of the incident pump beam projecting on *x* (*y*, *z*) direction. It has been demonstrated that **P**^(3*ω*)^ typically possesses the same polarization as that of the incident pump field^29^. However, **P**^(3*ω*)^ is zero when the applied pump field is circularly polarized^15^, which can also be explained from the perspective of angular momenta conservation in the nonlinear process^29,30^. These conclusions further solidify the polarization selectivity of third-order susceptibility.

We then theoretically analyze two typical cases of incident light polarization states. For Case 1, both incident beams are circularly polarized but with opposite chirality (depicted in Fig. 1 as *σ*^+^ and *σ*^−^). For simplicity, we only consider that the incident power of one beam is variable. Therefore the total synthesized electrical field (**E**_in_) of the two input beams can be expressed as ${{{\mathbf{E}}}}_{{{{\mathrm{in}}}}} \propto {{{\mathbf{E}}}}_{\sigma ^ + } + m{{{\mathbf{E}}}}_{\sigma ^ - }$, where ${{{\mathbf{E}}}}_{\sigma ^ + }$ (${{{\mathbf{E}}}}_{\sigma ^ - }$) is the electrical field of *σ*^+^ (*σ*^−^) circularly polarized input beam, and *m* is a variable that denotes the magnitude ratio of the electrical field of the *σ*^−^ and *σ*^+^ circularly polarized input beam. Note that the measured average power in the experiment of the variable incident beam is proportional to *m*^2^. In this case, the modulated THG intensity (when Δ*τ* = 0) can be expressed (see Section 1 of SI for details) as:

1 $$I^{\left( {3\omega } \right)} \propto {\it{\epsilon }}_0^2\chi _{11}^2m^2\left( {1 + m^2} \right)$$

For Case 2, one incident beam is linearly polarized, and the other beam is circularly polarized. Again, for simplicity, we consider that the circularly- (linearly-) polarized incident beam power is fixed (variable). Thus, the total synthesized electrical field is ${{{\mathbf{E}}}}_{{{{\mathrm{in}}}}} \propto {{{\mathbf{E}}}}_{\sigma ^ + } + m{{{\mathbf{E}}}}_{{{\mathbf{x}}}}$, where **E**_**x**_ is the electrical field of the horizontally polarized beam. In this case, the modulated THG intensity (when Δ*τ* = 0) can be expressed as:

2 $$I^{\left( {3\omega } \right)} \propto {\it{\epsilon }}_0^2\chi _{11}^2\left( {2m + m^2} \right)^2\left( {2 + 2m + m^2} \right)$$

From Eqs. (1) and (2), we can clearly see that the THG intensity can be fully modulated with the variable *m*^2^ (i.e., incident power of one incident beam) and the incident polarization states for both cases. For example, *I*^(3*ω*)^ is zero when *m* is zero (THG is absent with only one circularly polarized input), while *I*^(3*ω*)^ can be large when *m* is nonzero (THG is significantly modulated with the simultaneous presence of both input beams).

### Demonstration of chiral nonlinear optics modulation

**Fig. 2:**
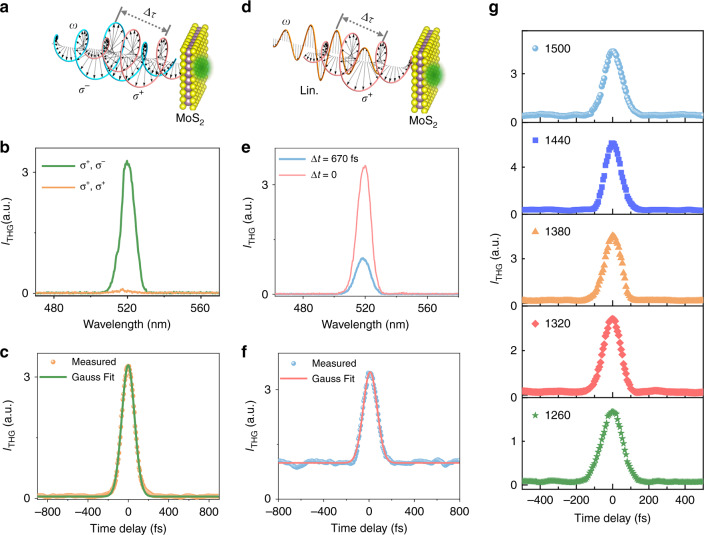
Coherent modulation of THG in monolayer MoS_2_. **a** Modulation scheme with two circularly polarized incident beams (Case 1). Purple and yellow spheres in MoS_2_ represent molybdenum and sulfide atoms, respectively. **b** Measured THG spectra with opposite (green curve) and identical circular polarization (orange curve) states at Δ*τ* = 0. **c** Measured THG signal as a function of the time delay for Case 1. **d** Modulation scheme with a linearly- and a circularly-polarized beam (Case 2). **e** Measured THG spectra when the linearly- and circularly-polarized beam with (blue curve) and without the time delay (pink curve). **f** Measured THG signal as a function of the time delay for Case 2. **g** Modulated THG signal as a function of the time delay with opposite circular polarizations at different wavelengths for Case 1.

We now experimentally verify the modulation principle (Fig. 1). Figure 2a illustrates the scheme of two incident beams both at the wavelength of ~1560 nm with identical power (~2 μW, corresponding to a pulse energy of 1 nJ) and opposite circular polarization states impinging on MoS_2_ (corresponding to Case 1 described by Eq. (1)). Detailed experimental setup and sample characterization are given in Section 2 of SI. Figure 2b shows the measured THG spectra in monolayer MoS_2_ when the spatially and temporally overlapped (i.e., Δ*τ* = 0) two incident beams possess the opposite (green curve) and identical (orange curve, for comparison) circular polarization, which generates strong and negligible THG signal. The very small (non-ideal zero) signal in the orange curve may be caused by the non-perfect circular polarization generated by the broadband (~1100–2000 nm) quarter-wave plate (QWP) used in our experiment. Figure 2c is the corresponding modulated THG signal as a function of Δ*τ*. Its full width at half maximum (FWHM) is ~130 fs, agreeing well with the THG autocorrelation results of the incident pulses with an FWHM of ~81 fs. This not only demonstrates that our THG modulation method can be used for the current autocorrelation-based pulse characterization method besides the modulation applications, but also shows the intrinsic ultra-fast speed (coherent process) of our modulation mechanism that is limited only by the incident pulse duration. Moreover, our all-optical modulation method is completely background free (i.e., the absence of THG plotted by the orange curve in Fig. 2b) because the incident light with the same chirality cannot generate THG^29,30^. The full background free characteristic is different from the typical collinear autocorrelation experiments where strong background noise is normally present^31^, or the recently reported SHG modulation approach where a polarization filter and carefully matching the light polarization with respect to the crystal axis of the material are needed^28^. A comparison of second- and third-order nonlinear susceptibilities and typical conversion efficiencies of typical 2D materials is also given in Section 9 of SI. The modulation depth (defined by the ratio of the sum of the maximum and minimum THG over the difference of the maximum and minimum THG) calculated from Fig. 2c is ~98.1%. Such a high modulation depth and ultra-fast response features make our approach promising for ultra-fast nanophotonics. We then analyze the conversion efficiency ~1.6 × 10^−7^% and the third-order susceptibility $\left| {\chi _{eff}^{\left( 3 \right)}} \right|$ ~1.7 × 10^−19^ m^2^/V^2^ when the incident beams are at ~1560 nm (Details about the calculation and at other incident wavelengths are given in Section 6 of SI), which are comparable to previous results (~10^−19^ m^2^/V^2^)^15,20,32,33^.

To further demonstrate the variability of this modulation approach, we also use one circularly polarized beam and one linearly polarized beam at ~1560 nm with the same power (~2 μW) impinging on MoS_2_ as schematically illustrated in Fig. 2d (corresponding to Case 2 and described by Eq. (2)). Figure 2e depicts the measured THG spectra without (Δ*τ* = 0, pink curve) and with (Δ*τ* = 670 fs, blue curve) time delay between the linearly- and circularly- polarized incident beams. The THG intensity is significantly enhanced when the two incident beams are synchronized in the time domain. Figure 2f gives the corresponding time-dependent THG signal with an FWHM of ~130 fs, which is also limited only by the incident pulse duration. Compared to Fig. 2c with two circularly polarized inputs (Case 1), the modulation is not fully background free because THG can be generated with the linearly polarized incident beam. However, it still possesses a significant modulation depth of ~55.7%.

The coherent modulation of THG enabled by optical chirality is governed solely by the *D*_3*h*_ symmetry of MoS_2_, which implies that this method can work at a wide operation bandwidth. Figure 2g shows broad THG modulation results at an operation wavelength range of ~1260–1500 nm when the two incident beams have opposite circular polarization states (Case 1 shown in Fig. 2a) and identical power (~2.5 μW, corresponding to a pulse energy of 1.25 nJ). Note that the availability of our laser wavelength limits our demonstrated bandwidth. In principle, our modulation concept can work at any wavelength due to its wavelength-independent nature.

**Fig. 3:**
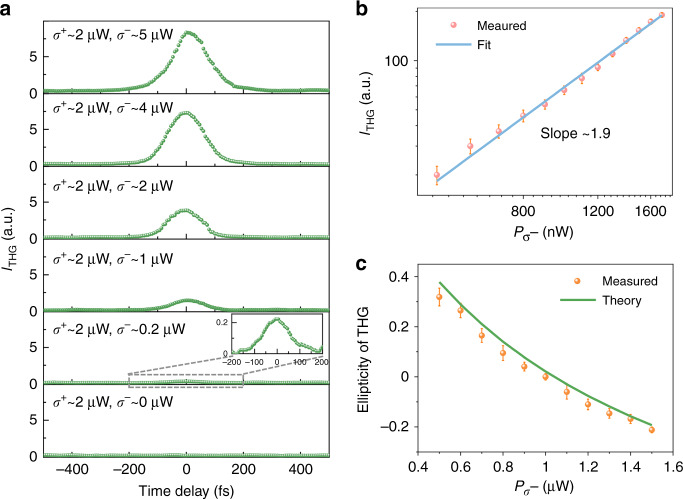
Modulated THG in monolayer MoS_2_ with two circularly polarized beams (Case 1). **a** Modulated THG signal as a function of the time delay under different incident powers. **b**, **c** Modulated THG’s power and ellipticity dependence on the *σ*^−^ circularly polarized incident beam power while the power of the *σ*^+^ circularly polarized beam is fixed at ~1 μW. Note that the measured average power of the incident beam is proportional to *m*^2^.

We next study the modulation performance dependent on the power and polarization of the two incident beams. For Case 1 of two circularly polarized beams discussed in Eq. (1) (as schematically illustrated in Fig. 2a), the power dependence of THG in monolayer MoS_2_ by changing the incident power of the *σ*^−^ circularly polarized beam is illustrated in Fig. 3a, while the incident power of the *σ*^+^ circularly polarized beam keeps at ~2 μW. As expected, the almost perfect modulation depth of ~100% (i.e., THG output is zero without the input of the *σ*^−^ circularly polarized beam or with a time delay between *σ*^−^ and *σ*^+^ circularly polarized beam) is demonstrated at different incident powers. Note that the intensity of THG is almost unchanged when the power of the *σ*^−^ circularly polarized beam changes from ~4 μW (corresponding to a pulse energy of 2 nJ) to ~5 μW (corresponding to a pulse energy of 2.5 nJ), which is caused by the saturation of the THG process in monolayer MoS_2_ due to (nonlinear) absorption^15,34^. When the power of the *σ*^+^ circularly polarized beam is ~2 μW, the THG modulation is observed with a minimum power of the *σ*^−^ circularly polarized beam ~0.1 μW (corresponding to a pulse energy of 50 pJ, Fig. S5). Therefore, we define the minimum working pulse energy as 50 pJ.

Figure 3b shows the THG dependence on the *σ*^−^ circularly polarized incident beam power in a double logarithmic plot. The slope of the experimental results (red dots) matches well with the theoretically calculated value of 1.92, which is obtained by plotting the THG intensity according to Eq. (1) versus *m*^2^ in the logarithmic coordinate. The deviation from the typical cubic power law between the incident beam power and THG intensity has never been reported before in monolayer MoS_2_. This comes from the fact that both incident beams contribute to the modulated THG signal. The demonstrated power law of the THG process offers a new degree of freedom to tune the THG process in an all-optical manner. Note that, as anticipated, the experimental power dependence of THG on the *σ*^+^ circularly polarized incident beam is the same as that of the *σ*^−^ circularly polarized incident beam.

Moreover, we analyze the polarization state of modulated THG by combining a QWP and a polarizer. Figure 3c shows the measured ellipticity of modulated THG as a function of the *σ*^−^ circularly polarized incident beam power. The red dots are measured results, and the blue curve is the theoretical calculation according to the definition of ellipticity $\sigma _{{{{\mathrm{THG}}}}} = \tan \left( {\frac{1}{2}\sin ^{ - 1}\frac{{1 - m^2}}{{1 + m^2}}} \right)$ see Section 1 of SI for the ellipticity calculation). The experimental results fit well with our theoretical calculation given by the measurement error (e.g., incident power fluctuation). Theoretically, the polarization of modulated THG can be infinitely close to *σ*^−^ or *σ*^+^ circular polarization when the variable *m* in Eq. (1) goes to infinite, but the exactly *σ*^−^ or *σ*^+^ circular polarization is forbidden by the conservation of angular momentum in the nonlinear process^29,30^ (details discussed in Section 5 of SI). Note that the dependence of modulated THG’s ellipticity on the *σ*^+^ circularly polarized incident beam shows the same variation trend but with an output opposite chirality.

**Fig. 4:**
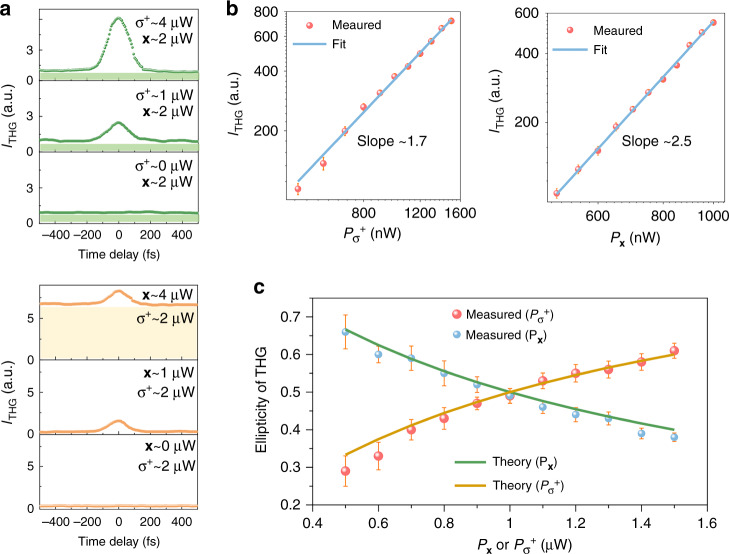
THG modulation performance in monolayer MoS_2_ with a linearly- and a circularly-polarized input beam (Case 2). **a** Modulated THG signal as a function of the time delay under different incident powers (upper panel: the power of the linearly polarized beam is fixed; lower panel: the power of the circularly polarized beam is fixed). **b** THG power dependence on the *σ*^+^ circularly- (linearly-) polarized beam while the power of the other beam with the linear (*σ*^+^ circular) polarization is ~1 μW. **c** THG’s ellipticity dependence on the *σ*^+^ circularly- or linearly-polarized beam while the power of the linearly- (*σ*^+^ circularly-) polarized beam is ~1 μW. Note that the measured average power of the incident beam is proportional to *m*^2^.

For Case 2 (one circularly polarized beam and one linearly polarized beam as described in Eq. (2) and Fig. 2d), the intensity variation of the modulated THG spectrum in monolayer MoS_2_ as a function of the time delay at different incident powers is illustrated in Fig. 4a. In the upper (lower) panel of Fig. 4a, the THG signal increases with the power of the *σ*^+^ circularly- (the linearly-) polarized beam when the power of the linearly- (the *σ*^+^ circularly-) polarized beam is fixed at ~2 μW. For the upper panel (lower panel), the background is almost fixed (increases significantly) and illustrated by the shaded green (yellow) area, which signifies an increasing (decreasing) modulation depth with the incident power. This is expected because the background comes from the linearly polarized incident beam’s THG. Nevertheless, we can adjust the modulation depth on demand for different applications (e.g., ~75.7%/~1.1% and ~48.3%/~87.5% in the top and middle rows of the upper/lower panel of Fig. 4a) by setting the incident power and polarization states. The power dependence of THG on the linearly polarized incident beam (blue line, the right panel of Fig. 4b) is determined by Eq. (2), which is fully confirmed by the measured results (Red dots). The left panel of Fig. 4b shows the power dependence of THG on the *σ*^+^ circularly polarized beam, agreeing well with the theoretical calculation (Section 1 in SI). The slope of the experimental results is ~1.7 (~2.5), which matches well with the theoretical calculation of ~1.8 (~2.5) obtained by plotting the THG intensity calculated in Section 1 of SI versus *m*^2^ in the logarithmic coordinate. The deviations from the typical cubic power law between the incident beam power and modulated THG signal are identical to what has been explained for Case 1 (Fig. 3b).

Further, the polarization of the output modulated THG signal is investigated for Case 2. Figure 4c shows the variation of ellipticity of the modulated THG with the incident power of the *σ*^+^ circularly- (linearly-) polarized input beam, where the dots are measured results, and the curves are theoretical calculations. The ellipticity decreases (increases) as the incident power of the linearly (*σ*^+^ circularly) polarized beam increases. Note that the ellipticity variation is not symmetrical with each other by changing the power of linearly- and *σ*^+^ circularly-polarized beam, which follows well with our theoretical calculation (Section 1 in SI).

## Discussion

In conclusion, we have demonstrated that symmetry enabled all-optical coherent modulation of THG with chiral light in monolayer MoS_2_ across a broadband wavelength range, which can be applied to other 2D materials and even bulk material (Fig. S3) with three-fold rotational symmetry. With two circularly polarized incident beams with opposite chirality, background free, perfect modulation depth (~100%), ultra-fast speed (~130 fs), and unlimited operation bandwidth are demonstrated. With a linearly polarized incident beam and a circularly polarized incident beam, variable modulation depth with ultra-fast speed (~130 fs) is accessed for different potential scenarios. Moreover, the output chirality of modulated THG signal and modulation performance can be tuned by changing the incident polarization and power. Our work opens a novel way to control the fundamental properties of THG (e. g., polarization, spin angular momentum, and intensity). The elliptically polarized (optical chirality) harmonics in the achiral medium possess spin angular momentum, which can explore spin-orbital coupling in the smallest possible systems. Further, the all-optical approach also provides a direct method for broadband ultra-short pulse characterization. The proposed method can be readily extended to high-order nonlinear processes, possibly stimulating the chiral high-harmonic generation for ultraviolet coherent light sources.

## Material and methods

### THG measurement

Figure S1 schematically shows the experiment setup for chiral THG modulation. The vertically polarized beam with a repetition rate of ~2 kHz from an amplified Ti:sapphire femtosecond laser system (Spectra-Physics Solstice Ace) is divided into two incident beams by a nonpolarizing beam splitter BS_1_ for the experiment. The spot size of the incident laser beam is ~2 μm. The transmitted beam goes through a delay line and is reflected by another nonpolarizing beam splitter BS_2_. The beam reflected by BS_1_ goes through a half-wave plate (HWP, AHWP10M-1600, ThorLabs) and then passes through BS_2_. The HWP changes the vertically polarized beam into a horizontally polarized beam. After BS_2_, the recombined vertically polarized and horizontally polarized beams are changed into *σ*^−^ and *σ*^+^ circular polarization by a QWP (AQWP10M-1600, ThorLabs). The spatially overlapped beams are synchronized in time by moving a homemade delay line, consisting of two mirrors mounted on a precision motorized translation stage (IKO LWLFG42B, SmartAct GmbH). The spatially and temporally overlapped beams are focused onto the sample by the objective lens obj. 1 (Nikon). The generated THG signal is collected by another objective lens obj. 2 (Nikon), which is delivered to a monochromator (Andor) equipped with a photomultiplier tube (Hamamatsu) connected to a lock-in amplifier (Stanford research system). Two 600-nm short pass filters (Edmund) after obj. 2 block the two incident beams. The ellipticity of the THG is analyzed by a homemade ellipticity analyzer consisting of a QWP (AQWP05M-600, ThorLabs) and a polarizer, which is placed behind the filter as shown in the inset of Fig. S1.

### MoS_2_ preparation and characterization

Monolayer MoS_2_ flakes are grown on a quartz substrate (a thickness of 500 μm) by chemical vapor deposition. First, a 5 mg/ml Na_2_MoO_4_ aqueous solution is spin-coated on the substrate, and then it is heated at 800 °C. Second, ~10 mg of sulfur is added and heated at 170 °C (5 min) under high-purity argon. Figure S2a gives the Raman spectrum of monolayer MoS_2_ excited by a laser at 532 nm. The Raman spectrum possesses two characteristic peaks representing the in-plane mode (E_2g_) and out-of-plane mode (A_1g_) at ∼384 cm^−1^ and ∼404 cm^−1^. The inset in Fig. S2a gives an optical image of a monolayer MoS_2_ flake on the quartz substrate. Figure S2b shows the measured photoluminescence spectrum (gray dots) of monolayer MoS_2_ excited by a laser at 532 nm. The Gauss fit curves show two characteristic peaks corresponding to the A-exciton (at ~675.5 nm, green curve) and B-exciton (at ~626.4 nm, orange curve), respectively.

## Supplementary information

SUPPLEMENTAL MATERIAL
