# Supplementary material for: Coherent modulation of chiral nonlinear optics with crystal symmetry
Source: Light Sci Appl. 2022 Jul 8;11:216. doi: 10.1038/s41377-022-00915-4 (PMC9270472; doi:10.1038/s41377-022-00915-4)
Supplement: Supplementary file 1 — SUPPLEMENTAL MATERIAL [file 41377_2022_915_MOESM1_ESM.docx]

Supplementary information

**Coherent modulation of chiral nonlinear optics with crystal symmetry**

**Yi Zhang**^1,2*^**, Xueyin Bai**^1^**, Juan Arias Muñoz**^1^**, Yunyun Dai**^1,3^**, Susobhan Das**^1^**, Yadong Wang**^1*^**, Zhipei Sun**^1,2*^

^1^Department of Electronics and Nanoengineering, Aalto University, Espoo 02150, Finland

^2^QTF Centre of Excellence, Department of Applied Physics, Aalto University, Espoo 02150, Finland

^3^Advanced Research Institute of Multidisciplinary Sciences, Beijing Institute of Technology, Beijing 100081, China

^*^email: [yi.1.zhang@aalto.fi](mailto:yi.1.zhang@aalto.fi); [yadong.wang@aalto.fi](mailto:yadong.wang@aalto.fi); [zhipei.sun@aalto.fi](mailto:zhipei.sun@aalto.fi)

**1. Third-order polarization and ellipticity calculation**

The third-order nonlinear process is determined by the third-order susceptibility $\chi_{ijkl}$, which is a fourth rank tensor determined by the symmetries of crystal lattices. The non-zero elements of the third-order susceptibility of MoS_2_ (belonging to *D*_3_*_h_* symmetry group^1,2^) include: $\chi_{yyyy}^{(3)}=\chi_{xxxx}^{(3)}=\chi_{yyxx}^{(3)}+\chi_{yxxy}^{(3)}+\chi_{yxyx}^{(3)}$, $\chi_{xxyy}^{(3)}=\chi_{yyxx}^{(3)}$, $\chi_{xyyx}^{(3)}=\chi_{yxxy}^{(3)}$, and $\chi_{xyxy}^{(3)}=\chi_{yxyx}^{(3)}$. The contracted matrix expression of $\chi_{ijkl}$ for materials belonging to *D*_3_*_h_* symmetry group can be written as^3,4^

$$\chi^{\left( 3 \right)}=\left[ \begin{matrix} \chi_{11} \\ 0 \\ 0 \end{matrix} \begin{matrix} 0 \\ \chi_{11} \\ 0 \end{matrix} \begin{matrix} 0 \\ 0 \\ \chi_{33} \end{matrix} \begin{matrix} 0 \\ \chi_{16} \\ 0 \end{matrix} \begin{matrix} 0 \\ 0 \\ \chi_{35} \end{matrix} \begin{matrix} \chi_{16} \\ 0 \\ 0 \end{matrix} \begin{matrix} 0 \\ 0 \\ \chi_{35} \end{matrix} \begin{matrix} {\chi_{11}}/3 \\ 0 \\ 0 \end{matrix} \begin{matrix} 0 \\ {\chi_{11}}/3 \\ 0 \end{matrix}\begin{matrix} 0 \\ 0 \\ 0 \end{matrix} \right]$$

where the first subscript (1, 2, and 3) denotes *x*, *y*, and *z*; and the second subscript denotes a combination of three components shown below

$$\begin{matrix} xxx \\ 1 \end{matrix} \begin{matrix} yyy \\ 2 \end{matrix} \begin{matrix} zzz \\ 3 \end{matrix} \begin{matrix} yzz \\ 4 \end{matrix} \begin{matrix} yyz \\ 5 \end{matrix} \begin{matrix} xzz \\ 6 \end{matrix} \begin{matrix} xxz \\ 7 \end{matrix} \begin{matrix} xyy \\ 8 \end{matrix} \begin{matrix} xxy \\ 9 \end{matrix} \begin{matrix} xyz \\ 0 \end{matrix}$$

Then, the third-order nonlinear polarization $\mathbf{P}^{\left( 3\omega\right)}$ of monolayer MoS_2_ can be expressed as

$$\left[ \begin{matrix} P_{x}^{\left( 3\omega\right)} \\ P_{y}^{\left( 3\omega\right)} \\ P_{z}^{\left( 3\omega\right)} \end{matrix} \right]=\epsilon_{0}\chi^{\left( 3 \right)}\left[ \begin{matrix} E_{x}^{3} \\ \begin{aligned} E_{y}^{3} \\ E_{z}^{3} \\ 3E_{y}E_{z}E_{z} \\ 3E_{y}E_{y}E_{z} \\ 3E_{x}E_{z}E_{z} \\ 3E_{x}E_{x}E_{z} \\ 3E_{x}E_{y}E_{y} \\ 3E_{x}E_{x}E_{y} \\ 3E_{x}E_{y}E_{z} \end{aligned} \end{matrix} \right]$$

where $\epsilon_{0}$ is the permittivity of free space, and *E_x_*, *E_y_*, and *E_z_* are the electric field components of the pump beam. We assume the pump beam satisfies paraxial approximation and *z*-components of the electrical field are negligible, the *x*- and *y*-component of $\mathbf{P}^{\left( 3\omega\right)}$ can be obtained

$$\left[ \begin{matrix} P_{x}^{\left( 3\omega\right)} \\ P_{y}^{\left( 3\omega\right)} \end{matrix} \right]=\epsilon_{0}\chi_{11}\left[ \begin{matrix} E_{x}^{3}+E_{x}E_{y}E_{y} \\ E_{y}^{3}+E_{x}E_{x}E_{y} \end{matrix} \right]$$

(1) When the two incident beams are circularly polarized (e.g., considering the right-handed (*σ* ^+^) (left-handed (*σ* ^–^)) circularly polarized beam with fixed (variable) incident power), the synthesized incident electrical field is $\mathbf{E}_{\mathrm{in}}\propto\mathbf{E}_{\boldsymbol{\sigma}^{+}}+m\mathbf{E}_{\boldsymbol{\sigma}^{-}}\propto\left( 1+m \right)\mathbf{E}_{\mathbf{x}}\boldsymbol{+}i\left( 1-m \right)\mathbf{E}_{\mathbf{y}}$, where $\mathbf{E}_{\boldsymbol{\sigma}^{+}}\propto\mathbf{E}_{\mathbf{x}}+i\mathbf{E}_{\mathbf{y}}$ and $\mathbf{E}_{\boldsymbol{\sigma}^{-}}\propto\mathbf{E}_{\mathbf{x}}-i\mathbf{E}_{\mathbf{y}}$ denote electrical fields of *σ* ^+^ and *σ* ^–^ circularly polarized beam, $\mathbf{E}_{\mathbf{x}}$ and $\mathbf{E}_{\mathbf{y}}$ denote the electrical fields of horizontally and vertically polarized beam, and *m* denotes the magnitude ratio of electrical field of *σ* ^–^and *σ* ^+^ circularly polarized beam. Note that the measured average power in the experiment of the variable incident beam is proportional to *m*^2^. The *x*- and *y*-components of $\mathbf{P}^{\left( 3\omega\right)}$ can be expressed as

$$\left[ \begin{matrix} P_{x}^{\left( 3\omega\right)} \\ P_{y}^{\left( 3\omega\right)} \end{matrix} \right]\propto\epsilon_{0}\chi_{11}m\left[ \begin{matrix} \left( 1+m \right) \\ \left( 1-m \right)i \end{matrix} \right]$$

As the THG intensity is proportional to the absolute square of third-order polarization $\mathbf{P}^{\left( 3\omega\right)}$, we can obtain the THG intensity (Eq. 1) in the main text $I^{\left( 3\omega\right)}\propto\epsilon_{0}^{2}\chi_{11}^{2}m^{2}\left( 1+m^{2} \right)$. The ellipticity of the THG signal can be calculated via Stokes parameters $S_{\boldsymbol{3}}=-2\mathrm{Im}\left[ P_{x}^{\left( 3\omega\right)}\left( P_{y}^{\left( 3\omega\right)} \right)^{*} \right]$^5,6^, where Im [⋅] denotes the imaginary part of a complex number. Thus, the ellipticity^5,6^ of the THG signal is $\sigma_{\mathrm{THG}}=\tan\left( \frac{1}{2}\sin^{-1}\frac{S_{3}}{S_{0}} \right)=\tan\left( \frac{1}{2}\sin^{-1}\frac{1-m^{2}}{1+m^{2}} \right)$.

(2.1) When two input beams are linear polarization (e.g., horizontal polarization) and circular polarization (e.g., *σ* ^+^ circular polarization), and power of the horizontally polarized incident beam is fixed. The synthesized input field is $\mathbf{E}_{\mathrm{in}}\propto\mathbf{E}_{\mathbf{x}}+m\mathbf{E}_{\boldsymbol{\sigma}^{+}}\propto\left( 1+m \right)\mathbf{E}_{\mathbf{x}}\boldsymbol{+}im\mathbf{E}_{\mathbf{y}}$, the *x*- and *y*-components of $\mathbf{P}^{\left( 3\omega\right)}$ can be expressed as

$$\left[ \begin{matrix} P_{x}^{\left( 3\omega\right)} \\ P_{y}^{\left( 3\omega\right)} \end{matrix} \right]\propto\epsilon_{0}\chi_{11}\left( 1+2m \right)\left[ \begin{matrix} \left( 1+m \right) \\ mi \end{matrix} \right]$$

Similarly, we can obtain the THG intensity $I^{\left( 3\omega\right)}\propto\epsilon_{0}^{2}\chi_{11}^{2}\left( 1+2m \right)^{2}\left( 1+2m+2m^{2} \right)$. The ellipticity of the THG signal is $\sigma_{\mathrm{THG}}=\tan\left\{ \frac{1}{2}\sin^{-1}\left[ \frac{\boldsymbol{2}m\left( \boldsymbol{1+}m \right)}{\boldsymbol{1+}{2m+2m}^{\boldsymbol{2}}} \right] \right\}$.

(2.2) When two input beams are linear polarization (e.g., horizontal polarization) and circular polarization (e.g., *σ* ^+^ circular polarization), and power of the circularly polarized incident beam is fixed. The synthesized input field is $\mathbf{E}\propto m\mathbf{E}_{\mathbf{x}}+\mathbf{E}_{\boldsymbol{\sigma}^{+}}\propto\left( 1+m \right)\mathbf{E}_{\mathbf{x}}\boldsymbol{+}i\mathbf{E}_{\mathbf{y}}$, the *x*- and *y*-components of $\mathbf{P}^{\left( 3\omega\right)}$ can be expressed as

$$\left[ \begin{matrix} P_{x}^{\left( 3\omega\right)} \\ P_{y}^{\left( 3\omega\right)} \end{matrix} \right]\propto\epsilon_{0}\chi_{11}\left( 2m+m^{2} \right)\left[ \begin{matrix} \left( 1+m \right) \\ i \end{matrix} \right]$$

Likewise, we can obtain the THG intensity (Eq. 2) in the main text $I^{\left( 3\omega\right)}\propto\epsilon_{0}^{2}\chi_{11}^{2}\left( 2m+m^{2} \right)^{2}\left( 2+2m+m^{2} \right)$. The ellipticity of the THG signal is $\sigma_{\mathrm{THG}}=\tan\left\{ \frac{1}{2}\sin^{-1}\left[ \frac{2\left( 1+m \right)}{2+{2m+m}^{2}} \right] \right\}$.

**2. Third harmonic generation measurement setup**


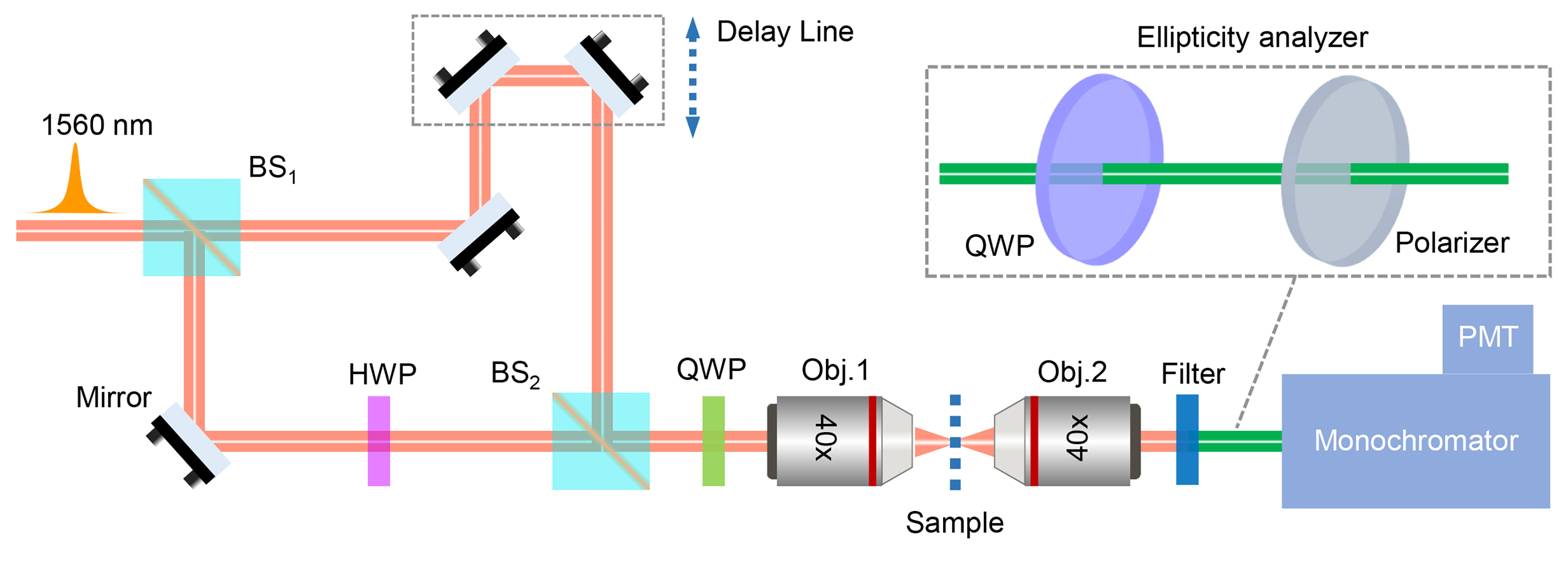


**Fig. S1.** **Experiment scheme for THG modulation with chiral light**. HWP: half-wave plate; BS: beam splitter; QWP: quarter-wave plate; obj.: objective lens; PMT: photomultiplier tube. The inset is a microscope image of monolayer MoS_2_ on SiO_2_. The inset is a homemade ellipticity analyzer consisting of a QWP and a polarizer.

**3. MoS_2_ preparation and characterization results**


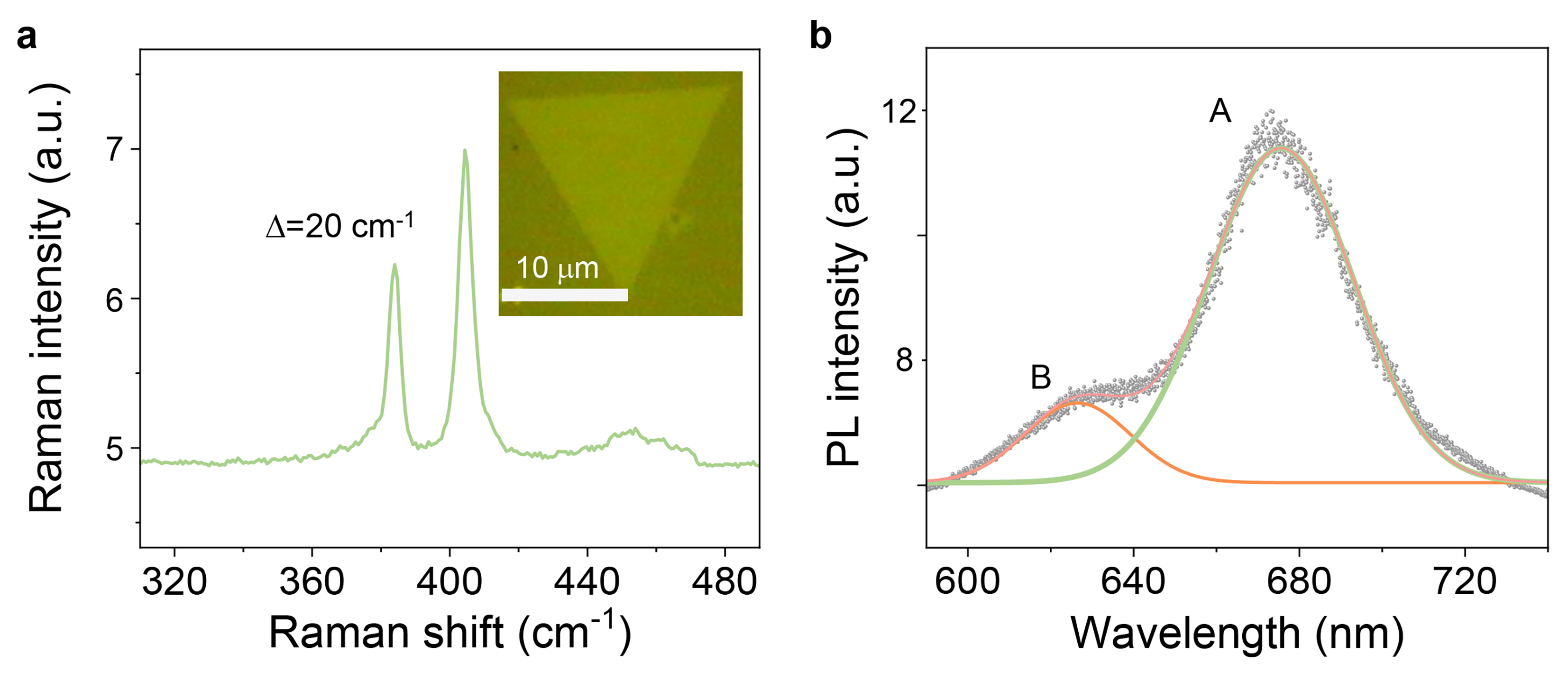


**Fig. S2** **Monolayer MoS_2_ characterization results**. **a** Raman shift of monolayer MoS_2_. The inset is a microscope image of monolayer MoS_2_. **b** Photoluminescence (PL) spectrum of monolayer MoS_2_. The Gauss fit curves with two peaks indicating the A-(green curve) and B-(orange curve) exciton, respectively.

**4. Modulation performance on Quarts**

Figure S3a illustrates the scheme of two incident beams both at the wavelength of ~1560 nm with identical power (~1 μW, corresponding to a pulse energy of 0.5 nJ) and opposite circular polarization states impinging on quarts (Case 1). Figure S3b shows the measured THG spectra in quarts when the spatially and temporally overlapped (i.e., Δ*τ* = 0) two incident beams possess the opposite (green curve) and identical (orange curve, for comparison) circular polarization, which generates strong and negligible THG signal. The very small (non-ideal zero) signal in the orange curve may be caused by the non-perfect circular polarization generated by the broadband (~1100-2000 nm) QWP used in our experiment. Figure S3c is the corresponding modulated THG signal as a function of Δ*τ*. Its full width at half maximum (FWHM) is ~130 fs, and the modulation depth is ~96.8%.

Figure S3d shows the scheme of one circularly polarized beam and one linearly polarized beam at ~1560 nm with the same power (~2 μW, corresponding to a pulse energy of 1 nJ) impinging on quarts (Case 2). Figure S3e depicts the measured THG spectra without (Δ*τ* = 0, pink curve) and with (Δ*τ* = 670 fs, blue curve) time delay between the linearly- and circularly- polarized incident beams. The THG intensity is significantly enhanced when the two incident beams are synchronized in the time domain. Figure S3f gives the corresponding time-dependent THG signal with an FWHM of ~130 fs. Compared to Fig. S3c with two circularly polarized inputs (Case 1), the modulation is not background-free because THG can be generated with the linearly polarized incident beam and the modulation depth is ~69.4%.


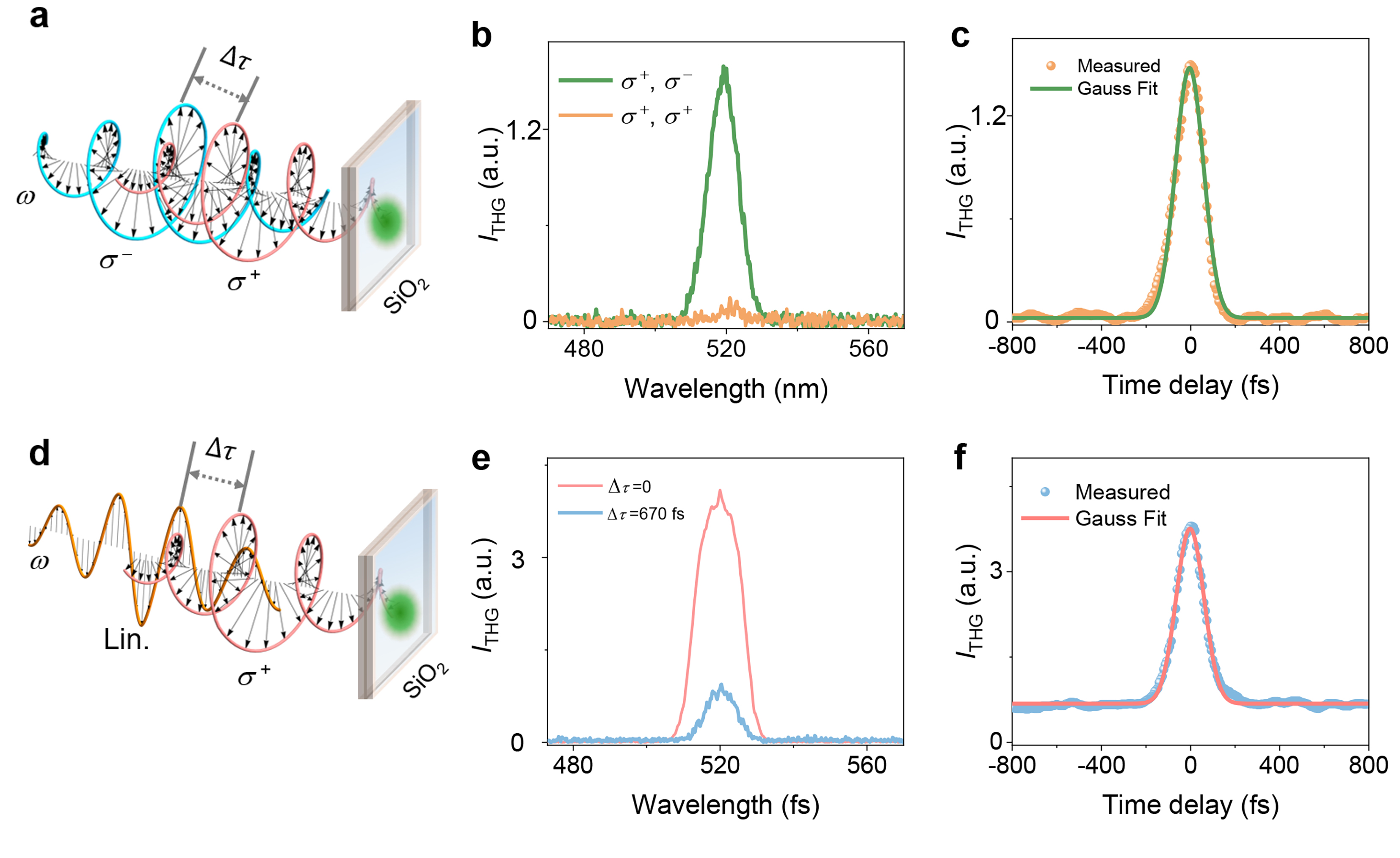


**Fig. S3 Coherent THG modulation in quarts. a** Modulation scheme with two circularly polarized incident beams (Case 1). **b** Measured THG spectra with opposite (green curve) and identical circular polarization (orange curve) states at Δ*τ* = 0. **c** Measured THG signal as a function of the time delay for Case 1. **d** Modulation scheme with a linearly- and a circularly-polarized beam (Case 2). **e** Measured THG spectra when the linearly- and the circularly-polarized beam with (blue curve) and without the time delay (pink curve). **f** Measured THG signal as a function of the time delay for Case 2.

**5. Angular momentum conservation in nonlinear processes**

THG is a coherent process and obeys the angular momentum conservation. Therefore, the THG governed by the three-fold rotational symmetry of a crystal structure can be expressed as $\sigma_{3\omega}\hbar-3\sigma_{\omega}\hbar=3N\hbar$^7,8^, where $\sigma_{3\omega}\hbar$ and $\sigma_{\omega}\hbar$ denote the spin angular momentum (corresponding to the chirality of the light beam, i.e., $\sigma^{\pm}=\pm1$) of the THG and fundamental beam, respectively; $3N\hbar$ denotes the angular momenta of the crystal lattice originating from the three-fold rotational symmetry. Therefore, there is no emitted THG photon when the fundamental photons have the same chirality^9^.

For the crystal structure with higher-order rotational symmetry, angular momentum conservation still holds for the THG process. For instance, the allowed THG process can be expressed as $\sigma_{3\omega}\hbar-3\sigma_{\omega}\hbar=4N\hbar$ for a crystal lattice with four-fold rotational symmetry. In this case, three fundamental photons with the same chirality can generate a THG photon with opposite chirality (i.e., the THG with opposite chirality compared to the fundamental wave is allowed), the modulation mechanism of our proposal would not work. Table S1 lists the allowed THG process determined by angular momentum conservation in crystals with different symmetry (e.g., three-, four-, five- and six-fold rotational symmetry), where “⊗” denotes the forbidden THG process. Therefore, our modulation mechanism applies to crystals with three- and six-fold rotational symmetry, in which the THG is forbidden with a circularly polarized pump beam.

**Table S1 THG process dependence on the crystal lattice symmetry**

| Crystal lattice symmetry | Angular momentum | | |
| --- | --- | --- | --- |
|  | Crystal lattice | Pump photons | THG photons |
| Three-fold rotational symmetry | $3N\hbar$ | $\pm3\hbar$ | ⊗ |
| Four-fold rotational symmetry | $4N\hbar$ | $\pm3\hbar$ | $\mp\hbar$ |
| Five-fold rotational symmetry | $5N\hbar$ | $\pm6\hbar$ | $\pm\hbar$ |
| Six-fold rotational symmetry | $6N\hbar$ | $\pm6\hbar$ | ⊗ |

**6. Calculation of third-order susceptibility**

To quantitatively describe the strength of the modulated THG in monolayer MoS_2_, we deduce the third-order susceptibility $\left| \chi_{eff}^{(3)} \right|$ following our previous work^10^, the $\left| \chi_{eff}^{(3)} \right|$ can be expressed as

$$\left| \chi_{eff}^{(3)} \right|=\frac{4\epsilon_{0}c^{2}}{3\omega d}\sqrt{n_{\omega}^{3}n_{3\omega}\frac{I_{3\omega}}{I_{\omega}^{3}}}$$

Where $\epsilon_{0}$ is the permittivity of free space, *c* is the speed of light in vacuum, *ω*, *I_ω_* and 3 *ω*, *I*_3_*_ω_* are the frequency, and peak intensity of the pump and THG pulses, *d* (~0.65 nm) is the thickness of monolayer MoS_2_. *n_ω_* and *n*_3_*_ω_* are the refractive index of the pump and THG pulses, respectively^11,12^. Note that we take Case 1 as an example, and the two incident beams have identical power. Thus, the *I_ω_* is the total peak intensity of the two incident beams.

Figure S4 shows the measured wavelength dependent $\left| \chi_{eff}^{(3)} \right|$ and conversion efficiency of THG in monolayer MoS_2_, which are comparable to previous results^10,13^. Note that in order to measure the power of the THG signal, we use a commercial femtosecond laser with parameters (e.g., repetition rate, wavelength) identical to the THG for the calibration. The power of the commercial femtosecond laser is first measured with a power meter and then measured by a spectrometer with photon counts. After this, we can obtain the power of the THG signal by just measuring the THG counts with the spectrometer.


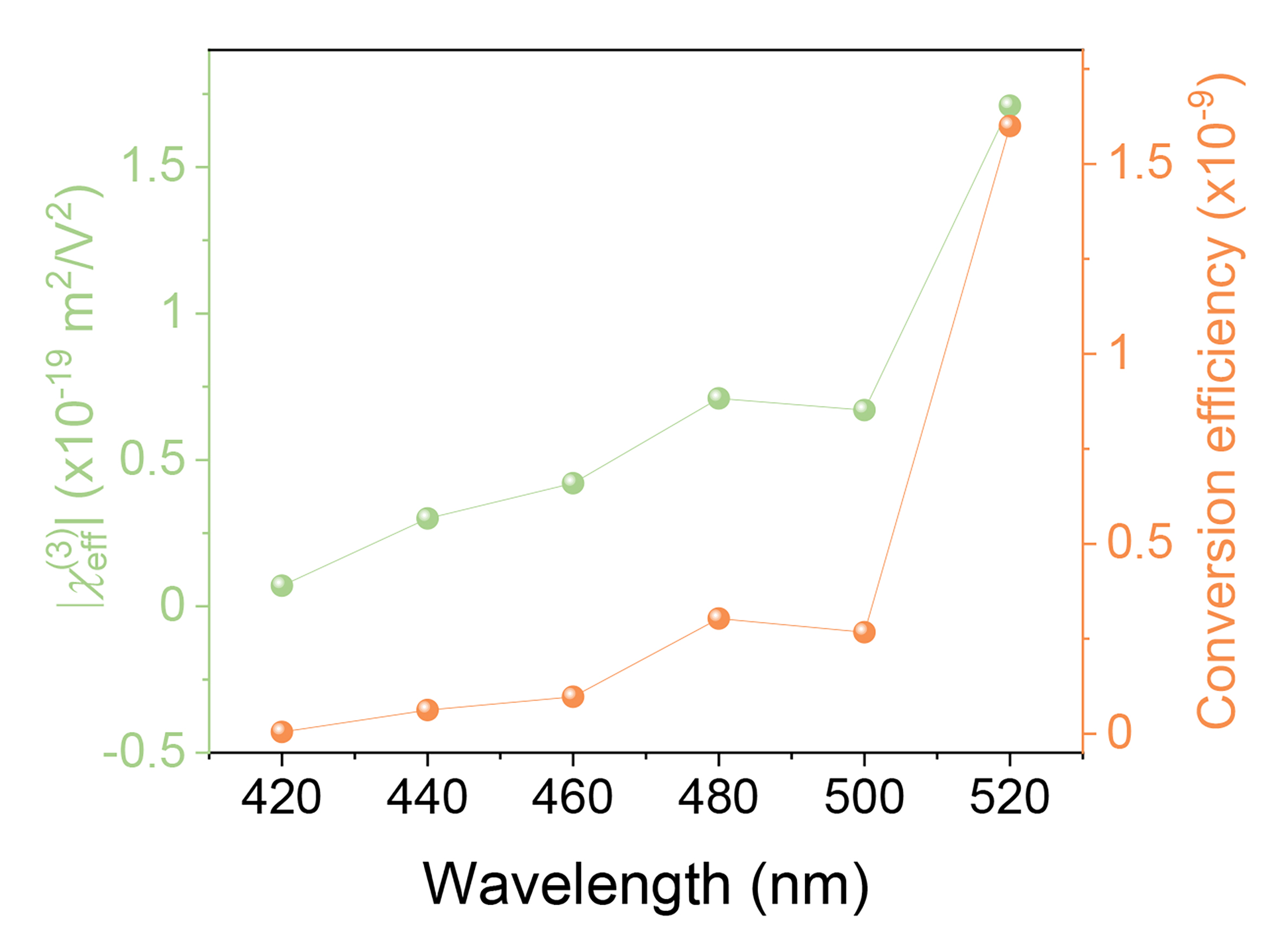


**Fig. S4 Wavelength-dependent third-order susceptibility and conversion efficiency of THG in monolayer MoS_2_.**

**7. Ellipticity of THG dependence on the power**

According to the theoretical calculation in Part 1, the THG output ellipticity is determined by the power of incident beams. Table S2 lists the THG ellipticity’s dependence on the incident beam power. In Figs. 2b, 2c, and 2g, the THG is linearly polarized due to the equal power of the *σ* ^–^ and *σ* ^+^ circularly polarized incident beams (Case 1). In Figs. 2e and 2f, the THG is elliptically polarized due to the unequal power of the *σ* ^–^ and *σ* ^+^ circularly polarized components produced by the linearly- and the circularly-polarized incident beams (Case 2). In Fig. 3a, the THG output is linearly (elliptically) polarized when the *σ* ^–^ and *σ* ^+^ circularly polarized beams have equal (unequal) power. In Fig. 4a, the modulated THG output is elliptically polarized due to the unequal power of the *σ* ^–^ and *σ* ^+^ circularly polarized components of the two incident beams.

**Table S2 THG polarization as a function of the input power** **under different incident polarization states**

| Cases | THG ellipticity |
| --- | --- |
| Case 1: Incident beams are circularly polarized but with opposite chirality [e.g., *σ* ^+^ and *σ* ^–^], the dependence of THG ellipticity on the *σ* ^–^ circularly polarized beam power when $P_{{}^{+}}$*~*1μW. | 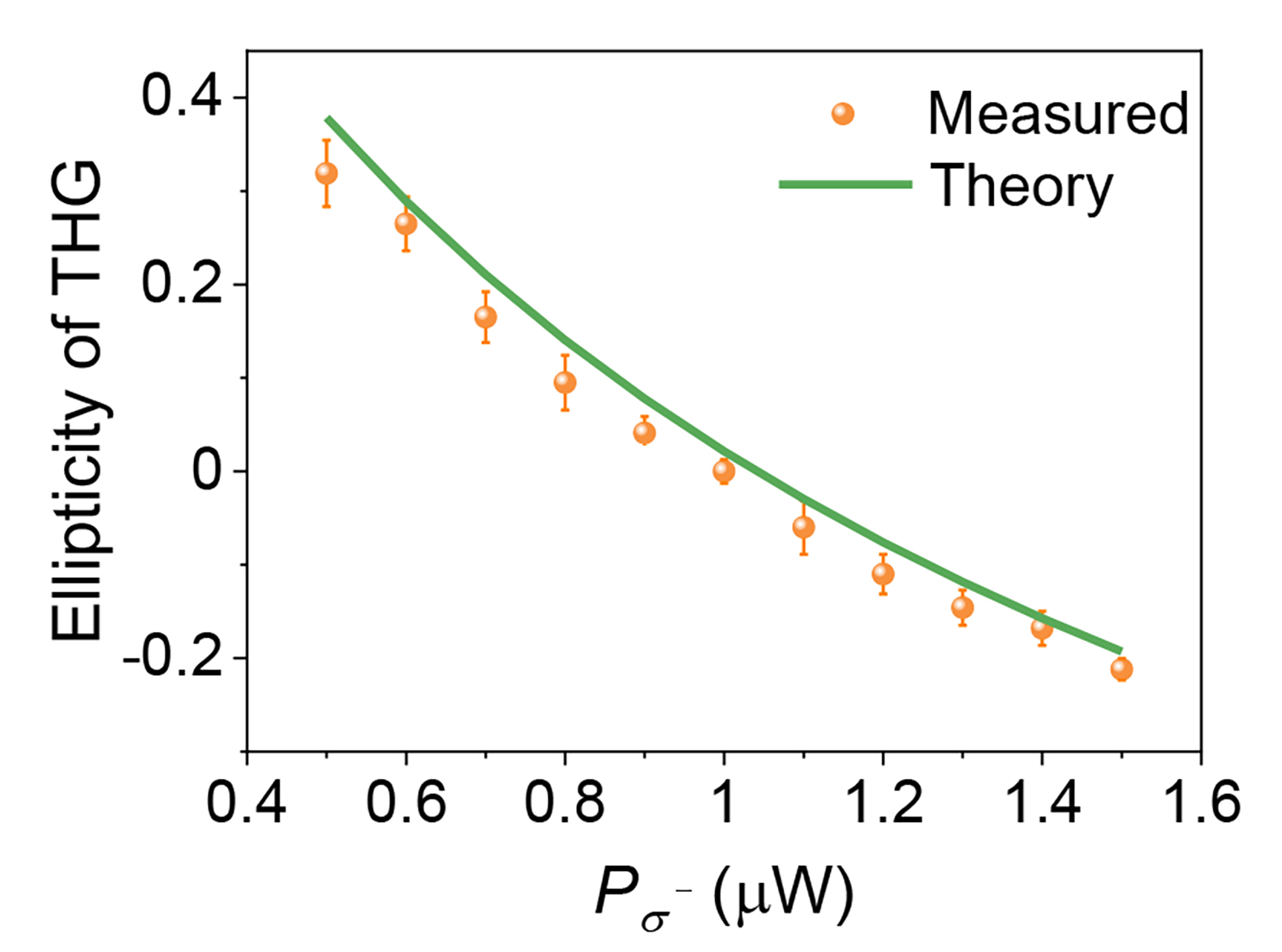  Fig. 3c |
| Case 2: Incident beams are a linearly polarized beam and a circularly polarized beam (e.g., **x** and *σ* ^+^), The dependence of THG ellipticity on the **x** (*σ* ^+^) polarized incident beam power when $P_{{}^{+}}$*~*1μW ($P_{x}$*~*1μW). | 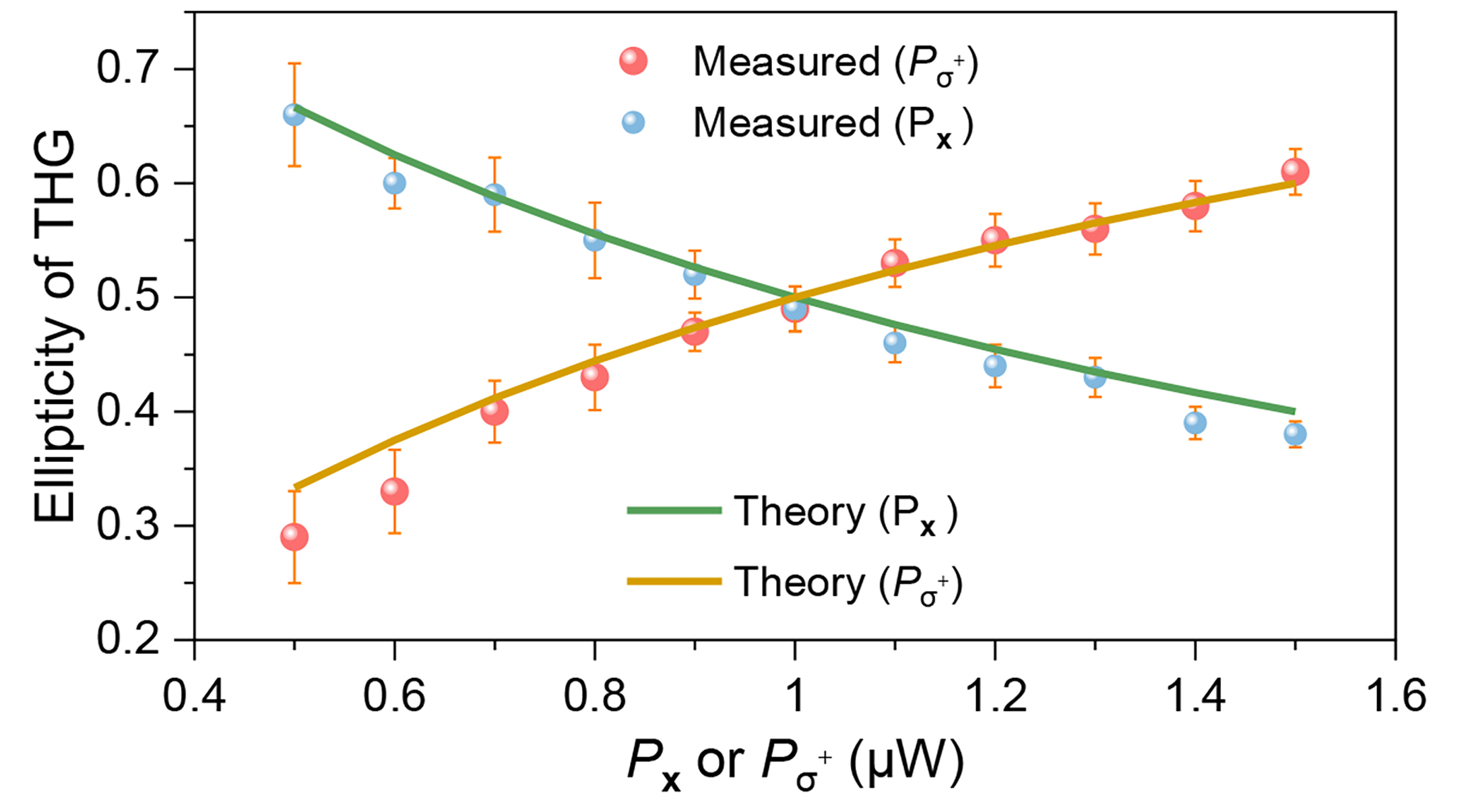  Fig. 4c |

8. Minimum working pulse energy

Figure S5 shows the modulated THG signal as a function of the time delay when the power *σ* ^–^ and *σ* ^+^ circularly polarized incident beam is *~*0.1 μW (corresponding to a pulse energy of 50 pJ) and *~*2 μW (corresponding to a pulse energy of 1 nJ), respectively.


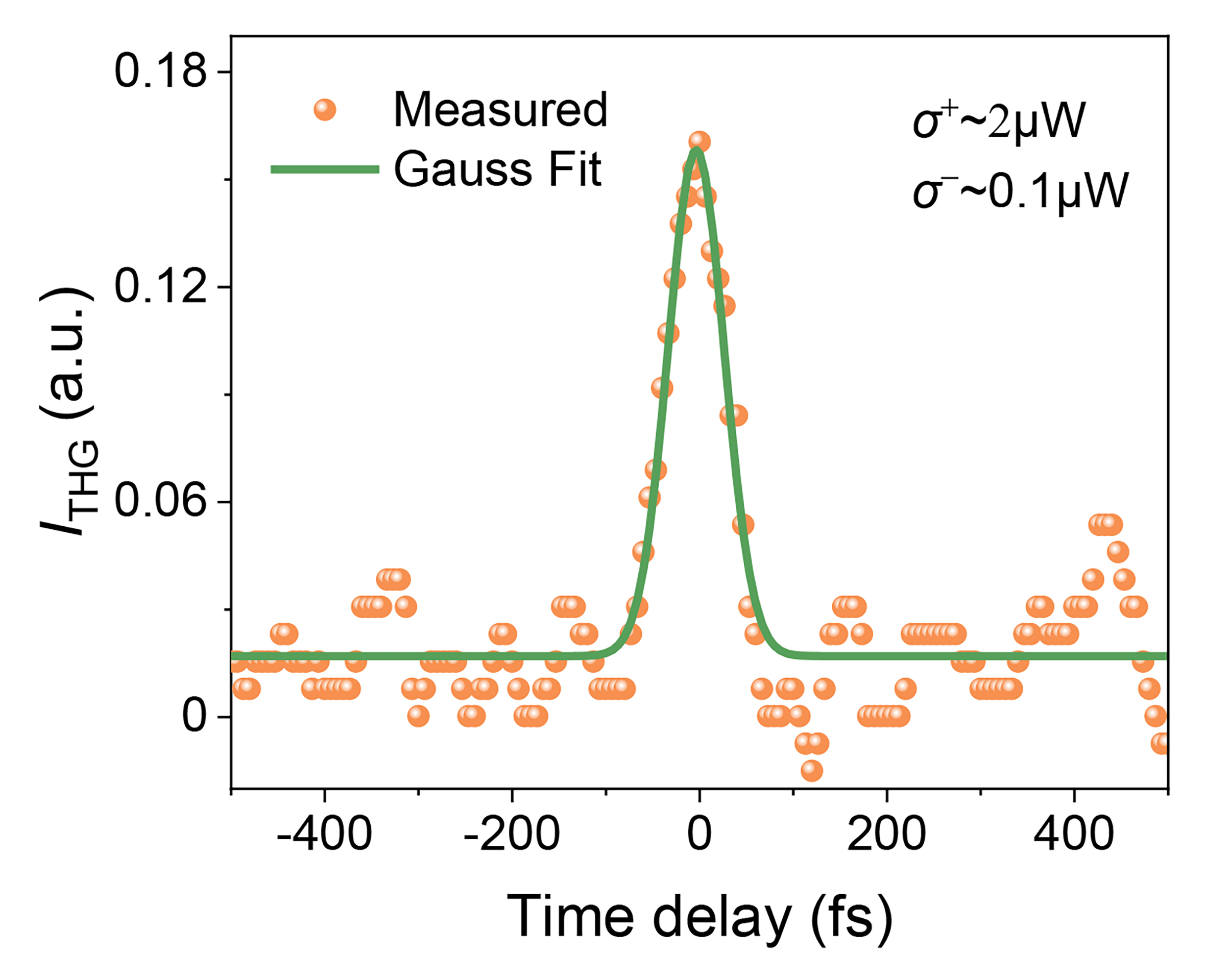


**Fig. S5 Modulated THG signal with two circularly polarized beams (Case 1).**

9. Comparison of nonlinear susceptibilities and conversion efficiency

Figure S6a gives a list of $\boldsymbol{\chi}_{\boldsymbol{eff}}^{\boldsymbol{(2)}}$ and $\boldsymbol{\chi}_{\boldsymbol{eff}}^{\boldsymbol{(3)}}$ comparison for the typical 2D materials. Fig. S6b shows the SHG and THG conversion efficiency of the same 2D materials in Fig. S6a, where most of the THG efficiency is surprisingly more efficient than that of SHG with a fundamental wavelength ~1560 nm.


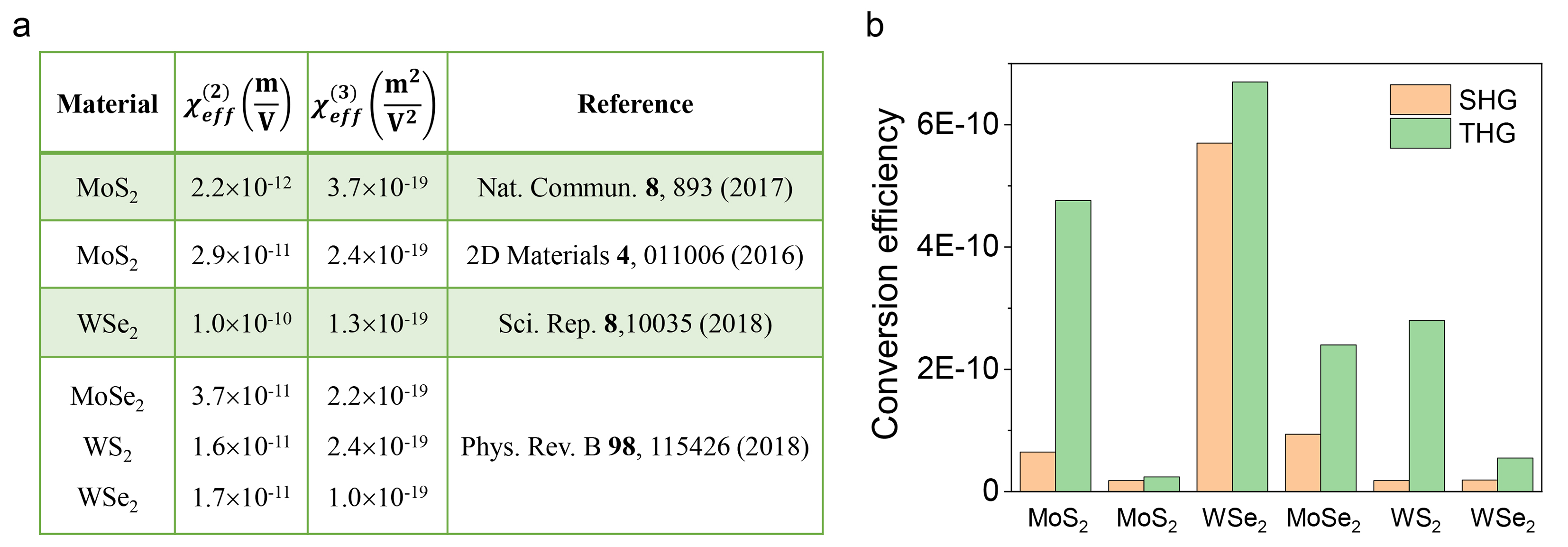


**Figure S6** **Comparison of reported nonlinear susceptibilities and conversion efficiency in 2D materials**. **a** Experimental $\chi_{eff}^{(2)}$ and $\chi_{eff}^{(3)}$ values of different 2D materials under pump wavelength ~1560 nm reported in the references. **b** SHG and THG conversion efficiency of the same 2D material in **a** from the same references.

References

1. Li Y. et. al. Probing symmetry properties of few-layer MoS_2_ and h-BN by optical second-harmonic generation. *Nano Lett.* **13**, 3329-3333 (2013).
2. Klimmer S., et al. All-optical polarization and amplitude modulation of second-harmonic generation in atomically thin semiconductors. *Nat. Photonics* **15**, 837-842 (2021).
3. Boyd, R. W. *Nonlinear optics*. Academic Press 2020.
4. Yang, X. L. & Xie, S. W. Expression of third-order effective nonlinear susceptibility for third-harmonic generation in crystals. *Appl. Opt*. **34**, 6130-6135 (1995).
5. Born, M. & Emil W., *Principle of Optics*, Elsevier 2013.
6. Berry, H. G., Gabrielse G. & Livingston A. E.. Measurement of the Stokes parameters of light. *Appl. Opt*. **16,** 3200-3205 (1977).
7. Simon, H. J. & Bloembergen N. Second-harmonic light generation in crystals with natural optical activity. *Phys. Rev.* **171**, 1104 (1968).
8. Yao, K., et al. Continuous wave sum frequency generation and imaging of monolayer and heterobilayer two-dimensional semiconductors. *ACS Nano* **14**, 708-714 (2019).
9. Cheng J., et al. Chiral selection rules for multi-photon processes in two-dimensional honeycomb materials. *Opt. Lett.* **44**, 2141-2144 (2019).
10. Dai, Y., et al. Electrical control of interband resonant nonlinear optics in monolayer MoS_2_. *ACS Nano* **14**, 8442-8448 (2020).
11. Yu, Y., et al. Giant gating tunability of optical refractive index in transition metal dichalcogenide monolayers. *Nano Lett.* **17**, 3613-3618 (2017).
12. Li, W., et al. Broadband optical properties of large-area monolayer CVD molybdenum disulfide. *Phys. Rev. B* **90**, 195434 (2014).
13. Wang Y., et al. Probing Electronic States in Monolayer Semiconductors through Static and Transient Third-Harmonic Spectroscopies. *Adv. Mater.* **34**, 2107104, (2022).
